# Hydroxycinnamic Acids and Derivatives Formulations for Skin Damages and Disorders: A Review

**DOI:** 10.3390/pharmaceutics13070999

**Published:** 2021-07-01

**Authors:** Marco Contardi, Martina Lenzuni, Fabrizio Fiorentini, Maria Summa, Rosalia Bertorelli, Giulia Suarato, Athanassia Athanassiou

**Affiliations:** 1 Smart Materials, Italian Institute of Technology, 16163 Genoa, Italy; martina.lenzuni@iit.it (M.L.); fabrizio.fiorentini@iit.it (F.F.); giulia.suarato@iit.it (G.S.); 2 DIBRIS, University of Genoa, 16145 Genoa, Italy; 3 Translational Pharmacology, Italian Institute of Technology, 16163 Genoa, Italy; maria.summa@iit.it (M.S.); rosalia.bertorelli@iit.it (R.B.)

**Keywords:** hydroxycinnamic acids, skin disorders, skin care, anti-oxidants, anti-inflammatory, anti-bacterial, biomaterials

## Abstract

Alterations of skin homeostasis are widely diffused in our everyday life both due to accidental injuries, such as wounds and burns, and physiological conditions, such as late-stage diabetes, dermatitis, or psoriasis. These events are locally characterized by an intense inflammatory response, a high generation of harmful free radicals, or an impairment in the immune response regulation, which can profoundly change the skin tissue’ repair process, vulnerability, and functionality. Moreover, diabetes diffusion, antibiotic resistance, and abuse of aggressive soaps and disinfectants following the COVID-19 emergency could be causes for the future spreading of skin disorders. In the last years, hydroxycinnamic acids and derivatives have been investigated and applied in several research fields for their anti-oxidant, anti-inflammatory, and anti-bacterial activities. First, in this study, we give an overview of these natural molecules’ current source and applications. Afterwards, we review their potential role as valid alternatives to the current therapies, supporting the management and rebalancing of skin disorders and diseases at different levels. Also, we will introduce the recent advances in the design of biomaterials loaded with these phenolic compounds, specifically suitable for skin disorders treatments. Lastly, we will suggest future perspectives for introducing hydroxycinnamic acids and derivatives in treating skin disorders.

## 1. Introduction

Skin is the tissue dedicated to the protection of the inner part of the body. It acts as a sensor for most of our sensations, such as the sense of temperature and pressure. This tissue can often undergo injuries, infections, or stresses, constantly being exposed to external agents and events [1].

Skin disorders and damages are emerging as one of the most troubling and challenging conditions for the healthcare systems in terms of rising cases, management, and correlated costs [2,3,4]. Acute and chronic wounds, diabetic ulcers, burns, psoriasis, and atopic dermatitis (AD) are the most diffused and alarming impairs of the skin tissue involving thousands of millions of people worldwide [5,6,7]. These conditions are connected with either genetic factors, metabolic alterations, environmental factors and lifestyle, injuries, or a combination thereof. Antimicrobial resistance is also playing a significant role in the development of secondary effects and in enhancing these alterations, aggravating the patients’ prognosis [8]. The concurrent abuse of soaps and hand disinfectants due to the COVID-19 pandemic might even support the onset of new disorders tightly connected with skin health and homeostasis modification. Indeed, the use of aggressive soaps and disinfectants can alter the equilibrium of the microbial environment, usually present in healthy skin, and modify the local pH, becoming a source of stress and irritation for the skin [9,10]. 

All these alterations are characterized mainly by the presence of a high concentration of free radicals and/or abnormal inflammatory response. For instance, after a burn event, a massive amount of reactive oxygen species (ROS) is generated. They can propagate the damage not only at the local but also to a systemic level, affecting other tissues and organs. This event recalls inflammatory cells, which can consequently contribute to increasing the ROS local and systemic generation. [11]. A similar scenario can be found in chronic diabetic ulcers, where the wound healing process is profoundly modified by the persistent hyper-glycemia condition. Here, a chronic inflammatory response is present, and an enormous quantity of inflammatory mediators and ROS are constantly released [12]. However, it is difficult to find a single molecular target due to the overlap of several altered molecular and metabolic pathways. Likewise, in dermatitis and psoriasis, the physiological alterations of the skin are attributed to upregulation of the release of pro-inflammatory mediators, such as chemokines and cytokines, requiring the use of anti-inflammatories such as corticosteroids [13,14]. In this scenario, natural compounds are gaining attention in the scientific and industrial communities due to their biocompatibility, anti-oxidant, anti-inflammatory, and antimicrobial properties [15,16,17,18]. Among these compounds, hydroxycinnamic acids and their derivatives have been explored in several fields such as food packing [19], cosmetics [20], pharmaceuticals [21], and nutraceuticals [22]. 

Biomedical nanotechnologies can offer great help in the improvement of phenolic stability, duration, bioavailability, and delivery at the site of action, to allow efficient exploitation of the compound’s therapeutic properties [23,24]. Currently, eligible biomaterials for this kind of application require several characteristics and should be selected case by case, depending on the different physio-pathological conditions that each skin injury or disorder presents.

Other reviews have already faced the importance of the hydroxycinnamic acids and derivatives for other specific applications such as cosmetics [20], their pharmacodynamics [25], pharmacokinetics [26], and toxicity [27]. Here, we will report on the newest sources and uses of hydroxycinnamic acids and derivatives, also in fields far away from the biomedical one, their potential employment for the treatment of some of the most diffused skin alterations, and how micro- and nano-technologies can help to speed up their introduction in the biomedicine world and ameliorate their efficacy.

## 2. Hydroxycinnamic Acids and Derivatives: General Uses and Current Applications

**Figure 1:**
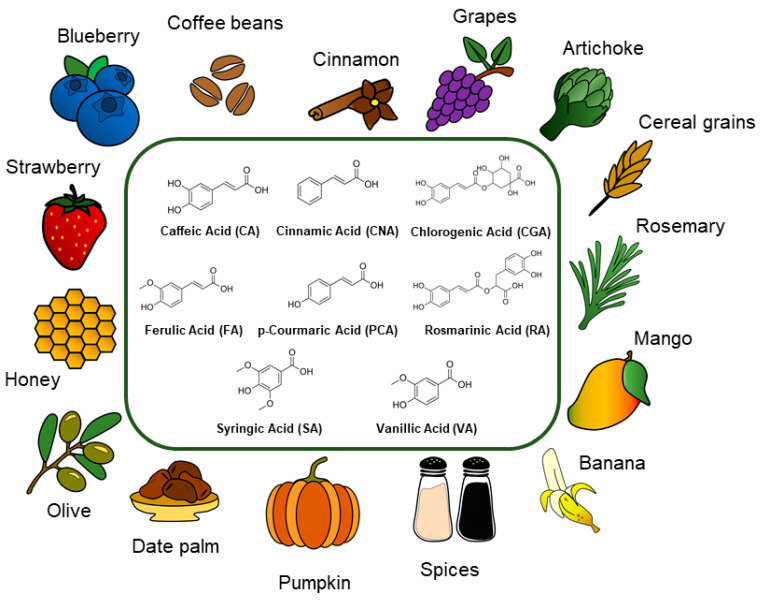
Schematic representation of the phenolic acids’ structures and their main source.

This class of compounds is synthetized via the shikimate pathway, a metabolic route used chiefly by plants but also fungi and algae. These molecules are involved in several functions inside the cells, which will be discussed in this section. In this review, we will focus our attention on cinnamic acid (CinAc), ferulic acid (FA), *p*-coumaric acid (PCA), caffeic acid (CA), vanillic acid (VA), syringic acid (SA), rosmarinic acid (RA), and chlorogenic acid (CGA). The chemical structures of these molecules are shown in Figure 1.

### 2.1. Cinnamic Acid

The term *cinnamic acids* defines a class of phenolic compounds comprising nine carbon atoms, naturally synthetized in plants (fruits and vegetables), with phenylalanine and tyrosine as precursors. Among these compounds, the most common and well-known are cinnamic acid, caffeic acid, ferulic acid, and *p*-coumaric acid, whose multiple nutraceutical benefits and bioactive properties have been known since ancient times. CinAc can be obtained from a variety of dietary resources, as shown in Figure 1, such as cinnamon (*Cinnamomum cassia* and *Cinnamomum verum J. Presl*), citrus fruits, spinach (*Spinacia oleracea* L.), cocoa (*Theobroma cacao* L.), grape (*Vitis vinifera* L.), tea (*Camellia sinensis* L. *Kuntze*), celery (*Apium graveolens* L.) [28], and other spices such as coriander, cloves, black pepper, and turmeric [29]. More recently, biotechnology and genetic engineering ensured the biosynthesis of CinAc and its alcohol and aldehyde derivatives via microorganisms such as *Escherichia coli, Saccharomyces cerevisiae, Pseudomonas putida,* and *Streptomyces lividans* [30]. As a pure compound, CinAc occurs as white crystalline scales with a melting point around 133 °C, it is slightly soluble in ethanol and other organic solvents, it is soluble in oils, but it is almost insoluble in water (logP = 2.13, water solubility 0.57 mg/mL).

Thanks to their photo-protective and antimicrobial action, CinAc compounds have found vast utilization in the food industry as nutraceutical supplements, food flavoring (in candies, chewing gums, mouthwash, and toothpaste), and packaging additives for food preservation [31]. In fact, CinAc is identified as a safe compound (GRAS, generally recognized as safe), by the U.S. Food and Drug Administration, and it has been used as an additive in aqueous vapor solutions employed in modified atmosphere packaging to limit microbial spoilage and preserve nutraceutical properties of fruits, without affecting the original flavors [29].

In addition to their applications in the food industry, CinAc and its derivatives have been widely explored in the pharmaceutical and biomedical field, displaying a broad spectrum of actions, such as anti-bacterial [32], anti-fungal, anti-cancer, anti-inflammatory, neuroprotective, cardioprotective, and anti-diabetic activities. In particular, considering the basic structure of CinAc, the benzene ring and the acrylic acid group allow various chemical modifications which lead to several CinAc synthetic derivatives, with enhanced bioactive properties, amplified electron-withdrawn ability (needed for an efficient free radical scavenging), modified lipophilicity, and ameliorated absorption and biodistribution [33,34]. Of particular interest are the positive effects exerted by CinAc on the diabetic pathology and its complications. Different mechanisms of action have been identified, including stimulation of insulin secretion, improvement of glucose tolerance, inhibition of protein glycation, and enhancement of pancreatic beta-cells functionality [28,35,36,37]. CinAc has also been widely used in cosmetics as a perfuming and flavoring agent, as well as a UV-absorbing and filtering molecule, an anti-oxidant, for skin/hair conditioning, or as an antimicrobial ingredient. Currently, the CinAc derivatives classified as UV filters, allowed in cosmetic preparations (for skincare and sunscreens), are ethylhexyl ester of 4-methoxycinnamic acid (octinoxate), isopentyl ester of 4-methoxycinnamic acid (amiloxate), 2-ethylhexyl ester of 2-cyano-3,3-diphenylacrylic acid (octocrylene), and 2-ethoxyethyl ester of 4-methoxycinnamic acid (cinoxate) [30]. In addition, CinAc has been investigated as a potential candidate for the treatment of skin hyperpigmentation. Kong et al. [38] studied the melanin biosynthesis profile within melanocytes and brown guinea pigs and observed a significant reduction (−29%) of melanin production in the melanoma mouse cells treated with 100 ppm of CinAc without any cytotoxicity. Moreover, the compound showed a depigmenting activity onto a model of UVB-induced hyperpigmentation via tyrosinase inhibition, thus acting as a skin-whitening agent.

### 2.2. Ferulic Acid

**Figure 2:**
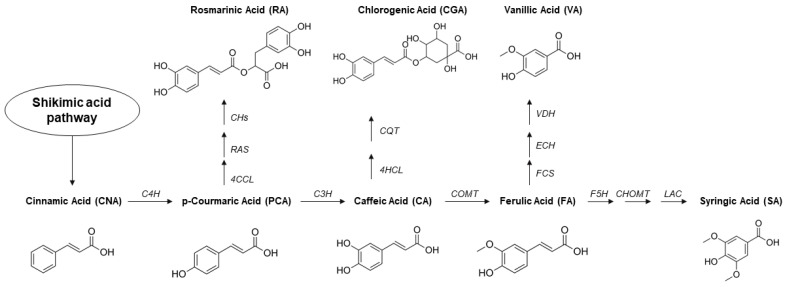
Schematic representation of the biosynthetic enzymatic shikimate pathway inside the plants for the phenolic acids production. Enzymes: 4CCL, 4-coumaryl-CoA ligase; 4HCL, 4-(hydroxy)cinnamoyl-CoA ligase; C3H, 4-coumarate 3-hydroxylase; C4H, cinnamate-4-hydroxylase; CHs, cytochrome P450-dependent hydroxylases; COMT, Caffeic acid 3-O-methyltransferase; CHOMT, caffeic acid 5-hydroxyferulic acid O-methyltransferase; CQT, hydroxycinnamoyl- CoA:D-quinate hydroxycinnamoyltransferase; ECH, Enoyl-CoA-hydratase; F5H, ferulate-5-hydroxylase; FCS, feruloyl-CoA-synthetase; LAC, laccase; RAS, hydroxycinnamoyl-CoA:hydroxyphenyllactate hydroxycinnamoyl transferase; VDH, vanillin-dehydrogenase.

Ferulic acid (named as 4-hydroxy-3-methoxycinnamic acid) is a natural phenolic compound that appears as a white crystalline powder and is present in many functional foods, including spinach, rice, citrus fruits, barley, carrots, and tomatoes (Figure 1). This molecule is the most abundant hydroxycinnamic acid in plants and is synthetized inside the shikimate molecular pathway [39]. The shikimic acid pathway is schematized in Figure 2. Like other hydroxycinnamic acids, FA plays a main role in the plant cell wall, affecting the color, response to pathogens, and mechanical properties [40]. The anti-oxidant potential of FA is usually attributed to its chemical structure, in particular to its phenolic nucleus and extended side chain. It forms a resonance-stabilized phenoxyl radical which gives it its potent natural activity. However, this compound is stable until 150 °C and is totally degraded at 226 °C [41]. FA has very low solubility in water (0.91 mg/mL, logP = 1.51) but it is soluble in ethyl acetate, dimethyl formamide, and alcoholic solvents such as ethanol, methanol, and isopropanol.

Nowadays, the functional properties of FA have been exploited and investigated in a wide range of applications. For instance, FA was widely utilized in food packaging and prevention due to the versatile capacities of this molecule. Indeed, several studies included this phenolic acid in polymeric systems for its strong anti-oxidant, anti-bacterialand UV-blocking properties; all features highly required and suitable for delaying food spoilage [42,43]. FA was introduced in a bioactive emulsion formulation as a natural pesticide against fungal parasites. Among the fungal parasites tested, the authors highlighted a superior efficacy of FA against *Botrytis cinerea*, which often infects wine grapes [44].

Han et al. [45] projected an electrode based on graphene-oxide and FA for the electrochemical determination of dopamine, a catecholamine highly present in the brain system and responsible for regulating various mechanisms in the human body.

As a biological agent, FA has been found active in different districts and diseases of the human body. For instance, this phenolic acid was demonstrated to be active in treating the oxidative stress and cell death induced by amyloid-beta, the protein responsible for the triggering of Alzheimer’s disease [46]. Always at the central nervous system level, it has been proved that FA can inhibit seizure activity, cognitive impairments, and thus, it can potentially be used in the therapy for epilepsy [47].

In diabetes, ferulic acid has been evaluated as a helpful tool for reducing the daily metformin dose due to its synergism in the glucose uptake and hypoglycemic effects of metformin [48].

Wang et al. [49] reported that FA could be employed as an anticoagulant agent to enhance the blood compatibility of silk fibroin. Similarly, Zhang and Shen [50] demonstrated the amelioration of the hemocompatibility and reduced platelet adhesion of a potential coating film based on poly (3-hydroxybutyrate-co-3-hydroxyhexanoate) for stent application. Finally, several other studies demonstrated anti-inflammatory, anti-arrhythmic, anti-cancer activities, hepato-, and eyes-protective properties [51,52,53,54,55].

### 2.3. p-Coumaric Acid

*p*-Coumaric acid (known as *p*-hydroxycinnamic acid) is synthetized in plants either from direct conversion of tyrosine or from oxidation of cinnamic acid as a metabolite of the shikimic acid pathway (Figure 2). As ferulic acid, this molecule plays an essential role in the regulation of the cell wall membrane. For this reason, it is diffused in several fruits, mushrooms, vegetables, and cereals (Figure 1). Its pristine form appears as a yellowish powder with a robust crystalline nature due to inter-/intramolecular interactions and, consequently, poor solubility in water (1.02 mg/mL, logP = 1.79). PCA is soluble in organic solvents such as dimethyl sulfoxide (DMSO), dimethyl formamide, and aqueous-ethanol solvent mixtures. This small molecule results in being stable from the thermogravimetric analysis until 170 °C and mostly degraded at 230 °C [41].

Currently, PCA is employed in various and different fields, primarily because of its anti-oxidant activity. Specifically, this phenolic acid was used as a food preservative capable of slowing down food alteration, protecting it from bacterial infection without affecting the food taste. With the same concept, PCA was included in polymeric film matrices to act as active agents for food packaging materials. An injectable poly-*ε*-caprolactone (PCL)-PCA biopolymer has been proposed and applied as a protective shield to treat coral wounds [56]. PCA has been electropolymerized to design an electrochemical sensor in order to monitor the presence of L-cysteine, a marker for different human health status, in biological fluids [57]. PCA was utilized as a grown regulator for the green microalga *Tetradesmus obliquus* BPL16, a promising source for biodiesel production [58]. In addition, bioengineered yeasts have been developed to scale up the synthesis and production of this compound [59].

As a biologically active agent, various effects have been attributed to PCA, paving the way for the application in cosmetics, nutraceutical, and pharmacology. In cosmetics, PCA has been proposed as a new natural ingredient for skin-lightening. Indeed, this phenolic acid is a tyrosinase inhibitor due to its chemical structure similar to l-tyrosine, the natural substrate for the synthesis of melanin [60,61]. As a dietary phenolic compound, PCA showed a protective anti-oxidant effect in colonic mucosa [62] and healing properties for gastric ulcers [63]. Together with FA and gallic acid, PCA increased the expression of anti-oxidant enzymes such as superoxide dismutase (SOD), glutathione peroxidase (GPX), and glutathione (GSH) at a cardiac level when orally administered in rats [64]. In addition, its action was investigated in different diabetic models in which the anti-diabetic effect was mostly justified by the upregulation of the expression of peroxisome proliferator-activated receptor γ (PPARγ) and the modulation of tumor necrosis factor α (TNF-α) and adipocytokine release [65]. Finally, PCA and its alkyl- and aryl-derivatives have been demonstrated active against *Leishmania braziliensis* and *Plasmodium falciparum*, the protozoa responsible, respectively, for leishmaniosis and malaria [66].

### 2.4. Caffeic Acid

Caffeic acid (known as 3,4-dihydroxycinnamic acid) is a secondary plant metabolite and one of the most abundant bioactive dihydroxycinnamic acids [67], and it consists of dihydroxyl and α,β-unsaturated (acrylic) carboxylic groups. This phenolic compound can be found in many food sources, as illustrated in Figure 1, such as coffee drinks, propolis, blueberries, apples, cider, potatoes, spinach, lettuce, cabbage, olive oil, wine, and tobacco leaves [68,69], and in some traditional medicinal herbs [70,71,72,73,74,75,76,77,78].

The plants can synthesize CA by the hydroxylation of *p*-coumaric acids through 4-coumarate 3-hydroxylase enzyme, as schematized in Figure 2. CA, as the other phenolic acids, is characterized by low solubility in aqueous solutions (less than 1 mg/mL, logP = 1.15), while it is soluble in ethanol, DMF, ethyl acetate, chloroform, and methanol. It appears as pale-yellow crystals or granules. The thermogravimetric analysis shows that CA undergoes thermal degradation at 241 °C, and it was demonstrated that the presence of this molecule could ameliorate the thermal stability of other materials [79,80]. High-temperature treatment and solvent extraction (with methanol and/or ethyl acetate) represent the main ways to extract CA from its sources, although a new environment-friendly approach was recently investigated by genetic engineering of an *E. coli* strain [81].

The excellent anti-oxidant activity allowed the applications of CA in different fields, such as in the fabrication of edible food packaging [82] and in the development of electrochemical sensors. Specifically, CA can be applied on nanocarbon composite electrodes to detect biological molecules involved in the progress of some diseases [83]. The anti-oxidant activity of CA was even exploited to decrease the oxidation of soybean biodiesel in the first period of storage [84].

This compound is also characterized by an antiviral activity, effectively inhibiting the in vitro replication of the influenza A virus in Madin-Darby Canine Kidney (MDCK) cells. This was possible thanks to a direct interaction between CA and some enzymes involved in the synthesis of new viral RNA copies [85].

### 2.5. Syringic Acid

Syringic acid (named as 4-hydroxy-3,5-dimethoxybenzoic acid) is a naturally occurring phenolic acid, synthesized by a series of enzymatic reactions involved in the shikimic acid pathway (Figure 2). It appears as a white shinning powder with a melting point around 208 °C, and it is soluble in ethanol, methanol, and ethyl ether; however, it is slightly soluble in water (1.42 mg/mL, logP = 1.04). SA is commonly found in various plants and fruits, as shown in Figure 1, including olives, walnut, cereals, dates, spices, pumpkin, grapes, honey, red wine, and vinegar [86]. Since phenolic compounds are an essential class of plant secondary metabolites, they are assumed to be involved in plant-plant and plant-microorganism interactions. Recent studies involving in vitro or soil tests have highlighted the impact of syringic acid in the alterations on soil rhizosphere microbial communities [87].

Recently, SA is gaining significant importance in the industrial sector. A laccase enzyme/syringic acid system, immobilized on a nylon membrane, was used as a model to develop a bioreactor for the bioremediation of vegetation waters polluted by phenolic compounds [88]. With a similar goal, photocatalytic ozonation of syringic acid, as phenolic wastewaters, was assessed onto titanium dioxide (photocatalyst) surfaces [89].

Several studies have reported the anti-oxidant, antimicrobial, anti-inflammatory, anti-diabetic, antiangiogenic, anti-hyperglycaemic, cardio-, hepato-, and neuroprotective properties for SA [86,90,91,92]. A high value of scavenging efficiency against the 2,2-diphenyl-1-picrylhydrazyl (DPPH^•^) free radical was revealed for SA when comparing different phenolic acids [93]. This compound has also shown anti-cancer and anti-mitogenic activities on human colorectal, breast, liver cancer, and leukemia cells [94,95,96]. In particular, such biomedical activities are assumed to rely on SA’s ability to eliminate ROS, which may cause cellular damage and carcinogenesis.

### 2.6. Vanillic Acid

Vanillic acid (known as 4-hydroxy-3-methoxybenzoic acid) is a phenolic derivative found in various edible plants and fruits, including açai berry, mango, vanilla pod, and ginseng (Figure 1). It is an oxidized form of vanillin used as a flavoring agent, and it is developed as an intermediate in the production of vanillin from ferulic acid, as shown in Figure 2. It appears as a white to light yellow powder or crystal with a melting point at 210 °C. Several reports have shown that vanillic acid is a highly unstable compound, rapidly prone to oxidation. It is observed that the anti-oxidant activity of free vanillic acid decreases from 24% to 10% after one week under constant environmental conditions [97]. VA is slightly soluble in water (1.28 mg/mL, logP = 1.43), yet it is soluble in organic solvents, such as ethanol and diethyl ether.

Several studies aiming at developing innovative films for active food packaging revealed that grafting natural phenolic acids such as VA might improve their anti-oxidant activity and stability [98,99]. Moreover, the oxidation mechanism of VA was used to develop an amperometric sensor for simultaneous determination of ascorbic acid, dopamine, and uric acid. In particular, here, VA was applied as an electroanalytical redox mediator [100].

The in vitro anti-oxidant mechanisms of VA include free radical scavenging activity, reductive power, and inhibition of lipid peroxidation [101,102]. The additional biological activities of VA include antimicrobial properties, as demonstrated by the growth inhibition of species and strains of *Listeria spp.*, anti-hypertensive, cardio- and hepato-protective activity, and chemo-preventive effects [102,103,104,105,106]. Recently, Ji et al. [107] investigated VA anti-diabetic and anti-inflammatory potentials on streptozotocin-challenged diabetes mellitus in rats. The authors demonstrated the protective properties of VA through lowering hepatic marker enzymes, reducing lipid peroxidation, altering carbohydrate metabolic enzymes, increasing anti-oxidant levels, altering protein profiles, and alleviating inflammation.

### 2.7. Rosmarinic Acid

Rosmarinic acid is an ester of caffeic acid and 3,4-dihydroxyphenyllactic acid. In nature it is biosynthesized starting from *p*-coumaric acid. RA can be found in various *Lamiaceae* medicinal plants (Figure 1), including rosemary, sage, basil, spearmint, perilla, and lemon balm [108]. It is a red-orange powder with a melting point at 171 °C, slightly soluble in water and well soluble in most organic solvents such as ethanol, DMSO, or dimethyl formamide (water solubility 1 mg/mL, logP = 2.57).

Recently, RA has found promising application in the design of food packaging materials. For example, edibles films containing RA were proposed for food packaging by Ge et al. [109], showing good water and UV resistance as well as strong mechanical stability. Additionally, RA and rosemary extract have been studied in various models and real foods to evaluate their ability as anti-oxidants to prevent food oxidation. Moreover, RA has been investigated for pigment stabilization/intensification of the color of beverages (e.g., juices and wines), containing unstable natural colorants, such as anthocyanins and carotenes [110]. In addition, RA was incorporated into micro/nanoparticles to overcome the limitations of phenolic acids in cosmetic formulations, such as poor skin retention and permeability [111,112]. Interestingly, RA is considered the most potent anti-oxidant among all hydroxycinnamic acid derivatives [113]. Aside from its anti-oxidant ability, RA and plant extracts containing RA have been shown in several research papers and patents to have a wide range of pharmacological and biological activities, including antitumor, antimicrobial, anti-diabetic, anti-inflammatory, immunomodulatory, cardio-, hepato- and neuro-protective properties [113,114]. From a molecular perspective, Jiang et al. [115] examined the effect of RA on the production of inflammatory mediators in lipopolysaccharides (LPS)-activated Raw 264.7 cells in order to unravel the potential anti-inflammatory mechanism of this compound. The results revealed that RA could inhibit high-mobility group box 1 protein, TNF-α, and IL-6 production, block nuclear factor kappa B (NF-κB) transcription factors, and inhibit the phosphorylation of nuclear factor of kappa light polypeptide gene enhancer in B-cells inhibitor alpha (p-IκB-α). These data indicate that the effect of RA is mediated by decreasing the levels of a wide range of pro-inflammatory mediators. Furthermore, Scheckel et al. [116] suggested that RA may be an inhibitor of the pro-inflammatory cyclooxygenase-2 (COX-2) expression in cancer and non-malignant mammary epithelial cells.

### 2.8. Chlorogenic Acid

Chlorogenic acid (defined as 3-(3,4-dihydroxycinnamoyl) quinic acid) is one of the most available molecules with respect to other phenolic acids due to its abundance in different foods and beverages, such as coffee. This cinnamate ester is obtained by condensing the carboxy group of trans-caffeic acid with the 3-hydroxy group of quinic acid. The biosynthesis of this molecule is described in Figure 2. It is an off-white powder with a melting point of 210 °C. CGA is soluble in organic solvents such as ethanol, DMSO, and dimethyl formamide and it has increased hydrophilicity (water solubility 40 mg/mL, logP = 0.370) compared to the other plant polyphenols. CGA can be naturally found in agricultural products (Figure 1), such as green coffee extracts, cocoa, citrus fruits, potatoes, berry fruits, apples, pears, and tea leaves [117,118]. CGA has been intensively studied for its use in packaging applications to extend the shelf-life of food products due to its excellent anti-bacterialand anti-oxidant properties. For example, Fu et al. [119] conjugated CGA onto gelatine and prepared a novel coating material for seafood preservation. Zhang et al. [120] used CGA to produce an intelligent food packaging material that can extend the food shelf-life and detect additives or contaminants. In the field of sensors, a thin film of CGA was used to modify a carbon-ceramic electrode in order to create an amperometric sensor for nicotinamide adenine dinucleotide hydride (NADH) determination [121]. CGA plays various important biological and therapeutic roles such as anti-oxidant, anti-bacterial, hepatoprotective, cardioprotective, anti-inflammatory, antipyretic, neuroprotective, anti-obesity, antiviral, antimicrobial, anti-hypertension, free radical scavenger, and even stimulant for the central nervous system [118,122,123,124,125]. As an example, it is reported that oxidative stress-induced secretion of IL-8 was significantly inhibited by CGA in Caco-2 cells [126]. In A549 human cancer cells, CGA induced up-regulation of cellular anti-oxidant enzymes and suppressed ROS-mediated NF-κB, activator protein-1 (AP-1) and mitogen-activated protein kinase (MAPK) activation [127].

## 3. Hydroxycinnamic Acids and Derivatives: Activities on Skin Disorders

**Figure 3:**
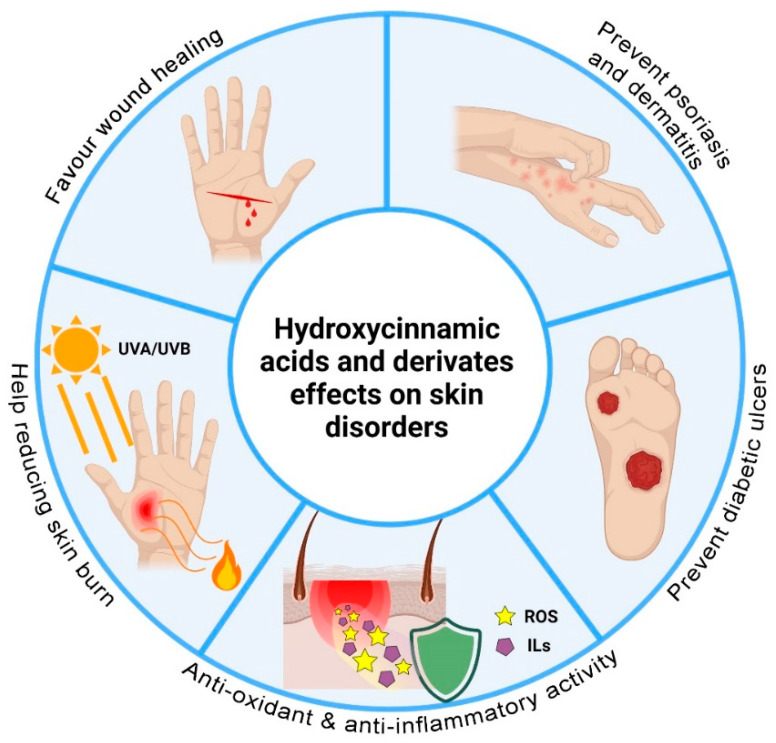
Schematic representation of the effects of the hydroxycinnamic acids and derivates on skin disorders created with BioRender.com (accessed on 15 May 2021).

Skin is the largest organ of the body and, as a consequence, it can be easily subjected to some damages, which alter the normal physiology of this important barrier. Wounds, burns, dermatitis, and psoriasis represent the main skin disorders. In this section, the potential of the hydroxycinnamic acid derivatives as pharmacological molecules in the treatment of skin diseases will be extensively discussed. The schematic representation of the various actions of hydroxycinnamic acids and derivatives toward skin disorders is illustrated in Figure 3.

### 3.1. Wound Healing

A wound represents an area in which skin has lost its function of physical and chemical barrier between the external environment and the inner part of the body. Immediately after the traumatic event, the body tries to solve this emergency situation through an elaborate cascade of events to restore the injured skin. In particular, wound repair consists of four main steps: haemostasis, inflammation, proliferation, and remodelling [128,129]. Haemostasis and inflammation occur immediately after the injury and take place to decrease as much as possible blood and fluids loss by activating the coagulation cascade, the inflammatory pathway, and the immune system cells [130]. When something does not work correctly in this early stage, the open tissue remains exposed to the external environment, increasing the probability of infections by pathogens. For instance, diabetic subjects are characterized by a delay in the healing process due to an overloaded inflammation step causing a high accumulation of neutrophils in the wound area [131]. This crowd of immune cells results in an abundant release of inflammatory molecules (e.g., cytotoxic enzyme and free radicals) that spread to the surrounding tissue damage [132]. Moreover, impairment of angiogenesis, alteration of the functions of keratinocytes and fibroblasts at epidermal and dermal levels, and factors related to collagen production are present, contributing to a delay in the healing process and generating a chronic wound environment. After the inflammation, the proliferation phase can start to form the granulation tissue, re-epithelialization, and neovascularization. The proliferation phase takes place after about 3 days from the initial wound and can last several weeks. It stimulates the proliferation and migration of fibroblasts and keratinocyte cells [128,133]. More specifically, fibroblasts are involved in the synthesis of the collagen needed to create a three-dimensional scaffold able to completely restore the wound area with new and healthy tissue. The remodeling phase can last from 6 months up to years. In this step, most of the previously involved cells, such as macrophages and endothelial cells, undergo apoptosis, and a long path of extracellular matrix (ECM) turnover occurs [129].

In this complex scenario, hydroxycinnamic acids and derivatives could act at different levels in the wound repair steps, even in chronic situations. Indeed, Ghaisas et al. [134] evaluated the activity of FA in an animal model of diabetic wounds. Topical and oral administration of FA determined the overall acceleration of the wound healing process. Evaluation of specific molecular targets, such as nitric oxide (NO), SOD, GSH, hydroxyproline, and hexosamine, confirmed the efficacy of FA in reducing inflammation, activating the anti-oxidant pathways, and promoting the formation of new skin tissue. Similarly, SA was evaluated in vivo for its wound healing potential on type 2 diabetic rat incisional wounds by Ren et al. [135]. The authors positively observed SA effects on blood glucose, lipid, and serum insulin levels, hydroxyproline content (as a measure of collagen content), and expression levels of several inflammation-related mRNAs and proteins (e.g., NF-κB, p65, TNF-α, IL-1β, IL-8, IL-10, and IL-2).

The ability of CA to stimulate the wound healing process was already investigated in the past, indicating that this bioactive molecule presented not only low cytotoxicity toward fibroblast cells [136] but also the tendency to increase their proliferation towards the repair of injured tissues [137]. Alexandru and co-workers [138] demonstrated that CA extracted from four medical plants stimulated collagen synthesis by fibroblast cells, confirming its high potential as a therapeutic agent in wound healing. CA also represents one of the active ingredients of the *Polygonum aviculare* extract, which was observed to promote the migration of keratinocyte and fibroblast cells, efficiently accelerating the epithelialization process of wounds in a murine model [77].

CGA is a well-described enhancer and promoter of the wound repair process. Indeed, in their works, Bagdas et al. [139,140] demonstrated that CGA could accelerate the wound healing process, enhance hydroxyproline and reduced-glutathione content, and decrease the malondialdehyde/NO levels in the wound bed without leaving any adverse effects on heart or kidney. In these works, the proliferative effect of CGA on fibroblasts, endothelial cells, and keratinocytes is associated with the regulation of collagen and matrix metalloproteinases secretion [139,140]. Moreover, CGA suppresses several inflammatory signaling pathways directly involved in the wound healing process, such as TLR4/MyD88/RELA pathway [141], JAT/STAT pathway [142], and nucleotide-binding oligomerization domain (NLRP3) inflammasome pathway [143], while it activates the anti-oxidant nuclear factor erythroid 2 (NFE2) [144] and MAPKs pathways [125,145].

Finally, several hydroxycinnamic acids and derivatives have been demonstrated as active antimicrobial agents [146]. Indeed, numerous works reported their efficacy against the main microorganisms responsible for wounds, burns, dermatitis, and psoriasis complications, such as *Pseudomonas aeruginosa* [147,148,149], *Staphylococcus epidermidis* [150], *Escherichia coli* [148,151,152,153], *Klebsiella pneumonia* [150], *Propionibacterium acnes* [154] and *Staphylococcus aureus* [155,156,157]. Having different antimicrobial mechanisms of action against bacteria, they could also be employed in combination with the current antibiotics utilized for skin disorder therapy in order to reduce the antibiotic consumption and improve the final antimicrobial effect.

Overall, thanks to some pivotal properties, such as high anti-oxidant activity, the ability to stimulate the skin cell proliferation, the synthesis of collagen, and the antimicrobial activity, hydroxycinnamic acid derivatives represent an optimal choice for the treatment of both acute and chronic (e.g., diabetic ulcers) wounds.

### 3.2. Burns and UV-Induced Damages

Overexposure to sunlight causes adverse effects on our skin, such as the generation of ROS and genetic mutations that can increase the risk of skin cancer. Burns are the short-term result of the effects of UV rays on the skin. Mild burns occur with an annual rate of 600/100,000 inhabitants, while severe burns have a rate of 5/100,000 inhabitants [158]. Over time, the signs of photoaging appear (e.g., degeneration of elastic fibers, skin dryness, and thickening), and the risk of carcinogenesis increases.

Several in vitro and in vivo studies have shown that hydroxycinnamic acids and their derivatives could protect the skin from the harmful effects of UV radiation by reducing inflammation, oxidative stress, and DNA damage. Specifically, Hseu and colleagues [159] assessed the trans-CinAc dermato-protective properties in a UVA-induced photoaging model. They observed that pre-treatment with 20–100 mM trans-CinAc significantly reduced ROS production in new-born foreskin cells subjected to UVA (3 J/cm^2^) stimulation, inhibited matrix metalloproteinase-1 (MMP-1) and MMP-3 upregulation by supporting nuclear translocation of nuclear factor erythroid 2-related factor 2 (Nrf2) (a regulator of cell resistance to oxidative stress). This anti-aging action restored proto-collagen production and protected mice’s skin integrity. A variety of studies demonstrated that RA could protect human epidermal keratinocytes (HaCaT) against ROS, generated either by H_2_O_2_ or by UVA/UVB exposure, providing convincing evidence that RA could play an important role in ROS scavenging and in decreasing the inflammatory response through a reduction of multiple pro-inflammatory mediators [160,161,162,163,164]. Furthermore, RA was demonstrated to attenuate UVB-induced macromolecular damages in human keratinocytes (e.g., protein carbonyl content and DNA strand breaks), exerting potent therapeutic and chemo-preventive effects related to UVB-induced carcinogenic processes [160,161]. In vivo experiments conducted by Sanchez-Campillo et al. [165] showed the ability of RA to inhibit mice cutaneous alterations caused by UVA exposure and other ionizing radiations (“skin photo-carcinogenesis”). According to the authors, the photo-protective activity of RA is exerted through free radical scavenging, regulation of tyrosinase activity, and stimulation of melanin production.

Regarding skin photo-carcinogenesis, the research conducted by Feng et al. [127] provided molecular evidence for the anti-carcinogenic potential of CGA; in particular, the effect of CGA on 12-*O*-tetradecanoylphorbol-13-acetate(TPA)-induced cell transformation was investigated on a skin epithelial JB6 P+ cell line. The results indicated that pre-treatment of JB6 cells with CGA blocked UVB- or TPA-induced activation of AP-1 and NF-κB transcription factors, which are known to play essential roles in promoting skin tumors. Specifically, AP-1 is a major regulator of the expression of the COX-2 and MMP-1 enzymes, which play critical roles in UV-mediated skin inflammation and photoaging. Previously, another study had shown that oral administration of CGA before exposure to γ-radiation significantly reduced in vivo chromosomal damage [166].

Her et al. [167] observed that topical application of *Aronia melanocarpa* extract, which is rich in CGA and rutin, protected mouse dorsal skin from damage, following UVB irradiation, by attenuating collagen disruptions. Furthermore, Ha et al. [168] found that *Rhus javanica* extract (RJE), which includes SA as one of its most abundant phenolic compounds, attenuated UVB-induced photodamage and inflammation by modulating the expression of MMPs and COX-2 proteins in SKH-1 hairless mice. In particular, among the compounds in the RJE, SA exhibited the most potent inhibitory effect in HaCaT cells on the transactivation of the AP-1 protein. Similar to what was found previously by Ha et al., Kwon et al. [169] confirmed the attenuation of UVB-induced photoaging by *Spatholobus suberectus* stem extract, which has syringic and vanillic acid as major active components, via modulation of MAPK/AP-1/MMPs signaling pathway in HaCaT cells. Likewise, researchers demonstrated that CA protects human dermal fibroblasts (Hs68 cells) against UVB-induced photo-toxicity in a dose-dependent manner by downregulating the activation of the UVB-induced MAPKs and NFκB signalling pathways. In particular, CA significantly inhibited the expression and release of MMP-1, reduced UVB-induced ROS production, and collagen degradation in UVB-irradiated Hs68 cells [170]. Moreover, the extract obtained from the plant *Tragopogon graminifolius*, rich in polyphenolic compounds, including CA, was seen to stimulate the wound closure successfully when topically administrated on second-degree burn wounds in a rat model [75].

As previously introduced, several MMPs isoforms, such as MMP-2 and MMP-9, are known to be overexpressed in UVB-irradiated skin tissues and contribute to the acceleration of photoaging and the development of skin cancer. Staniforth et al. [171] analyzed the suppressive effects of FA on UVB radiation-induced MMP-2 and MMP-9 activities in mouse skin. Histological analyses showed that FA attenuates the degradation of collagen fibers, abnormal accumulation of elastic fibers, and epidermal hyperplasia induced by UVB. Using in vivo studies, Peres et al. [172] found that a sunscreen formulation with FA amplified the sun protection factor by about 37% and the UVA protection factor by 26%. Lastly, SA treatment has been shown to prevent cutaneous carcinogenesis when used on UVB-treated HaCaT and, in vivo, SA pre-treatment of SKH-1 hairless mice skin significantly suppressed UVB-induced skin tumour incidence in a dose-dependent manner. More in detail, SA showed to exert a potent chemo-preventive activity in skin photo-carcinogenesis mainly by inhibiting the Nox/ROS/PTP-κ/EGFR signaling pathway [173].

### 3.3. Dermatitis and Inflammation

Dermatitis is an inflammatory condition of the skin which includes atopic, allergic, and irritant contact, seborrheic, and stasis dermatitis. Among them, atopic dermatitis is the most diffused, affecting 10–20% of children and 1–3% of adults in developed countries. AD is a long-lasting chronic inflammation state of the skin strictly connected with asthma and allergic episodes. The origin of this condition has to be sought in a simultaneous presence of barrier skin defects and dysregulations of the immune system response [14]. Dysfunction of the degradation mechanism of filaggrin proteins and alteration of ceramides composition leads to increased permeability and, consequently, dehydration of the skin. Furthermore, impairment of cell-mediated immunity induces hypereosinophilia, with high levels of immunoglobulin E (IgE), histamine, and inflammatory mediators such as IL-4, IL-5, IL-13, IL-31, and TNF-α [174]. The combination of these alterations produces episodes of pruritus, flares, xerosis, and skin cracked that typically appear in AD patients. Moreover, xerosis state has been demonstrated to be a prone environment for bacterial outbreaks of *Staphylococcus aureus*, requiring additional antibiotic administration [175].

In this scenario, phenolic acids can play a potential role in the design of new therapies for this condition due to their multifunctionality. Indeed, FA was proved to be active in an induced AD mouse model, reducing the expression of cytokines such as IL-4, IL-6, TNF-α, and IL-31, and repressing T helper type 2 cells (Th2) immune-mediated response [176]. Also, the CA derivate caffeic acid phenethyl ester (CAPE) manifested a high anti-inflammatory activity in atopic dermatitis, suppressing the generation of pro-inflammatory cytokines and the production of NF-κB protein in keratinocyte cells [177]. Jang et al. [178] induced atopic dermatitis-like lesions in NC/Nga mice by 2,4-dinitrofluorobenzene (DNFB), followed by RA application, which was found to suppress the levels of IL-4, interferon-γ (IFN- γ), and total serum IgE. Furthermore, RA significantly inhibited skin lesions and ear thickness development in DNFB-treated NC/Nga mice. Lee et al. [179] conducted clinical studies to evaluate the effect of RA-based emulsions on patients suffering from AD and found an improvement of skin manifestations (e.g., erythema, pruritus, and dryness) in treated patients. Cho et al. [180] assessed the potential therapeutic effect of CGA, the main active component in *Pyrus ussuriensis Maxim.* leaves extract, on the progression of AD in in vitro and in vivo experimental models. The authors observed how the CGA present in the extract could ameliorate AD-like symptoms by suppressing the pro-inflammatory cytokines (particularly IL-6 and IL-1β) and immune stimuli in NC/Nga mice treated with 2,4-dinitrochlorobenzene (DNCB). Moreover, CGA significantly ameliorated the dermatitis severity, scratching tendency, and trans-epidermal water loss compared to the control. The anti-inflammatory activity of CA was also evaluated towards acute and chronic irritant contact dermatitis on a mouse model, determining an inhibition effect due to the ability of CA to down-regulate the synthesis of TNF-α, IL-6, and IL-1β cytokines [67].

Going towards a general inflammatory condition, the ability of these phenolic acids to modulate the inflammatory response was also tested in other in vitro and in vivo skin models. For instance, Song et al. [137] evaluated the anti-inflammatory action of CA, which was able to inhibit histamine release in RBL 2H3 mast cells, and, at the same time, both metillin-induced [^3^H] arachidonic acid (AA) and prostaglandin E_2_ (PGE_2_) production in Raw 264.7 macrophage cells. CA tested on Raw 264.7 cells could suppress Interleukin-1 Receptor Associated Kinase 1 (IRAK1) and IRAK4, two enzymes directly involved in the inflammatory response, because of their role in the production of COX-2, TNF-α, and NO [181]. Bùfalo and co-workers [182] highlighted the capacity of CA to inhibit the NO production from macrophage cells and to suppress the LPS-induced signaling pathway. In another study, the authors have demonstrated the CA ability in decreasing the expression of two transcriptional factors involved in the inflammation process (NF-κB and COX-2) in mouse skin [183].

VA is also involved in the inhibition of oxidative stress, pro-inflammatory cytokine production (e.g., TNF-α, IL-6), and NFκB activation. In addition, VA has been shown to inhibit LPS-induced COX-2 expression and PGE2 production by mouse peritoneal macrophages in vitro [184]. The mechanisms by which VA exerts its anti-inflammatory effects were confirmed in vivo in murine models of inflammatory pain (i.e., carrageenan-induced paw oedema model) by Calixto-Campos et al. [185]. Vanillic acid and syringic acid were also the major identified phenolic acids in *Cucumis melo* L. fruits, which Moustafa et al. [186] studied to assess their potential anti-inflammatory and anti-ulcer activities on carrageenan-induced rat paw oedema. The fruit pulp extracts showed a significant decrease in oedema volume and anti-ulcer activity (i.e., ulcer number and severity) after 4 h compared to the standard drugs.

Similar effects were observed for RA by Rocha et al. [187] and Usha et al. [188] in an in vivo model of local inflammation lowering IL-1β and TNF-α levels.

Dos Santos et al. [189] evaluate the anti-inflammatory, analgesic, and antipyretic activities of CGA in rats. In comparison to control, CGA decreased carrageenin-induced paw oedema volume starting from the second hour of the experimental procedure. The authors stated that such activities might be derived from the inhibitory action of CGA in the synthesis/release of inflammatory mediators, such as TNF-α and NO, involved in these responses. A similar result was reported by Ha et al. [190] when topically applied an *Artemisia capillaris* extract, rich in CGA, reduced dermatitis scores, haemorrhage, hypertrophy, and hyperkeratosis of the epidermis in the dorsal skin and ears of mice. This treatment also decreased the plasma levels of histamine (1.5 fold) and IgE (1.4 fold) compared to the control.

### 3.4. Psoriasis

Psoriasis is a chronic skin disorder whose hallmarks are erythematous and scaly plaques, mainly localized at the scalp, the lumbosacral area, the joints, and the limbs. It affects about 2–3% of the world’s population, with a prevalence of young adults (15–25 years old); moreover, 10–30% of the affected people can also develop psoriasis arthritis at later stages. The rapid reoccurrence and the peculiar appearance may have sociological implications on patients’ everyday lives, thereby causing significant morbidity [191]. Although governed by the patient’s immune system, the intricate pathophysiology of psoriasis has been attributed to several factors, such as genetic predisposition and environmental triggers (e.g., trauma, emotional stress, infections). The skin lesions, covered with white and silver scales, are the result of (a) dysregulated proliferation of keratinocytes, (b) limited epidermal turnover time, (c) proliferation of new dermal blood vessels, and (d) infiltration of multiple inflammatory cells. The vicious cycle is sustained by the keratinocytes-mediated stimulation of proinflammatory chemokines and cytokines, which ultimately impair the barrier function of the epidermal layer and cause its thickening [192]. In the past decades, various pathogenic mechanisms have been identified, leading to different therapeutic approaches with improved patient compliance and limited adverse effects. Usually, treatments for psoriasis management comprise topical therapy (with vitamin D analogues, corticosteroids, retinoids in the form of gels, creams, and ointments), systemic therapy (with orally delivered immunosuppressants such as methotrexate and cyclosporine), phototherapy, and biological agents (such as monoclonal antibodies, gene therapy, anti-cytokine treatments, anti-T cells therapy, phosphodiesterase 4 inhibitors) [191].

Among the molecules under study, RA resulted in being the most investigated for psoriasis.

The first evidence of its efficacy was reported by Zhou et al. [193]. In their work, the authors induced an inflammatory condition in keratinocytes by using a synthetic agonist of the TRL-3 receptors, triggering inflammatory cytokine expression. The administration of RA was demonstrated to statistically reduce the levels of IL-6, IL-8, IL-1β, TNF-α, NF-κB in this induced inflammatory reaction of epidermal keratinocytes, suggesting a potential efficacy of RA for psoriasis. More recently, Zhang and colleagues [194] elucidated a possible mechanism of action for RA. Indeed, the phenolic acid was able to regulate the JAK2/STAT3 signaling pathway, inhibiting the IL-23/Th17 (T helper type 17 cells) axis, which plays a significant role in psoriasis. Similar outcomes have been observed by Koycheva et al. [195], who demonstrated the efficacy of pure RA and *Lavandula angustifolia Mill.* extract (rich in RA) in interfering in the JAK1/STAT2 signaling pathway, down-regulating the gene expression of NF-κB and affecting the PI3K/AKT signaling. All these molecular pathways are typically altered in the psoriasis condition.

FA was also described as a potential therapeutic molecule for psoriasis. Lo et al. [196] studied, via docking and in vivo psoriasis mice models, the efficacy of FA in affecting the IL-17A/IL-17RA interaction and its capacity to reduce the skin thickness and inflammation after oral administration in mice.

Recently, various plant-based therapeutics have been effectively employed for the psoriasis treatment, such as extracts from *Aloe vera*, *Curcuma longa*, *Mahonia aquifolium*, *Matricaria recutita*, *Silybum marianum*, *Gaultheria procumbens*, and *Thespesia populnea*, which acted reducing TNF-a, IL-8, IL-12, and p65 expression levels [191].

Cheng et al. investigated the effect of vanillin, which is converted to VA in the liver, on imiquimod (IMQ)-induced psoriatic skin inflammation in mice. The proposed treatment significantly improved IMQ-induced histopathological changes of skin in a dose-dependent manner and significantly decreased both the amounts of IL-17 and IL-23 as well as the infiltration of immune cells in the skin tissues of IMQ-treated mice [197].

In a different study, the therapeutic potential of the ethanol extract of *Artemisia capillaris*, which contains chlorogenic acid, coumarins, and flavonoids as major components, was verified for psoriasis treatment in HaCaT cells and IMQ-induced psoriasis-like mouse models. The IMQ-mouse models with topical application of this extract exhibited lower epidermal thickness and intracellular adhesion molecule-1 (ICAM-1) expression level. In addition, the treatment of psoriasis-like lesions inhibited the hyperproliferation of keratinocytes and the infiltration of leukocytes [198].

While oral therapies are frequently associated with adverse effects as a consequence of high systemic dosages, conventional topical treatments generally exhibit poor permeation through the hyper-keratotic and rigid psoriatic scales. Therefore, multiple and frequent applications are needed to obtain a therapeutic effect, thus causing possible skin irritation. To overcome these limitations and improve the patient response, advanced nano-formulations (such as solid lipid nanoparticles, nanostructured lipid particles, polymeric nanoparticles, and nano-emulsions) have been explored as psoriasis drug delivery systems [191,192]. Nanocarriers with optimized size, shape, surface charge, and functionalities (hyaluronic acid, heparin sulfate, or chondroitin sulfate with affinity towards skin receptors) have shown an enhanced permeation through the stratum corneum by loosening the tight junctions between the epithelial cells and delivering their bioactive molecules into the skin deeper layers. Based on a more efficient targeting ability and permeation enhancement, innovative nanotechnology-based delivery systems hold great potential in psoriasis therapy, improving the benefits–risks ratio [192]. Examples of these approaches will be discussed in detail in Section 4 of this review.

## 4. Hydroxycinnamic Acids and Derivatives: Advanced Formulations

**Figure 4:**
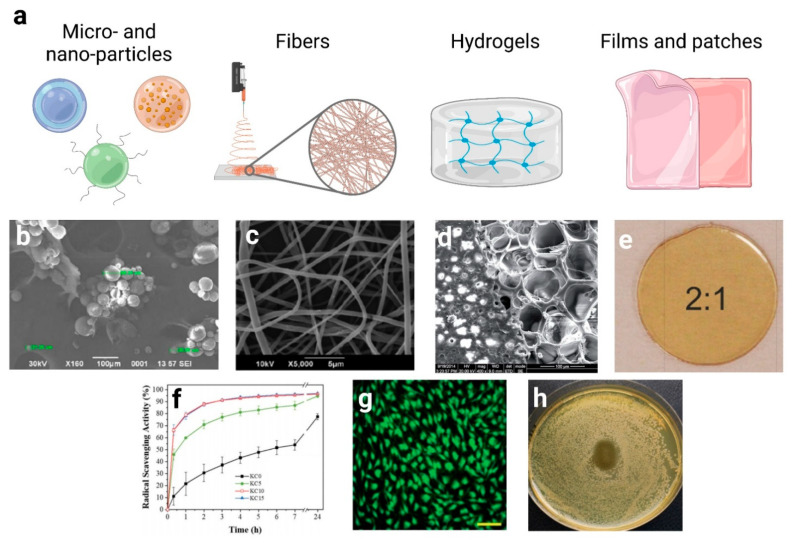
(**a**) Schematic representation of the principal nanocarriers exploited in the treatment of skin disorders created with BioRender.com (accessed on 15 May 2021). (**b**) SEM image of phyto-vesicles made of soy lecithin and cholesterol loaded with rosmarinic acid. [208] Copyright^®^2020 with permission from Elsevier. (**c**) SEM image of PVP/keratin nanofibers loaded with cinnamon essential oil [213]. This article is licensed under a Creative Commons Attribution-NonCommercial 3.0 Unported Licence, Published by The Royal Society of Chemistry. (**d**) Surface/cross-section SEM image of caffeic acid/gelatin-based hydrogels. Adapted from [229] with permission from The Royal Society of Chemistry. (**e**) Photograph of a transparent PVP/PCA-based film with a weight ratio 2:1. Adapted from [243] with permission from The Royal Society of Chemistry. (**f**) Anti-oxidant capacity of PVP/keratin/cinnamon essential oil-based nanofibers in a radical scavenging assay. [213] This article is licensed under a Creative Commons Attribution-NonCommercial 3.0 Unported Licence, Published by The Royal Society of Chemistry. (**g**) Fluorescence image of NIH 3T3 fibroblast cells adhering and proliferating inside a caffeic acid/gelatin-based hydrogel. Adapted from [229] with permission from The Royal Society of Chemistry. (**h**) Anti-bacterialefficacy of a PVP/PCA-based film against *Escherichia coli*. Adapted from [243] with permission from The Royal Society of Chemistry.

Poor solubility in water, crystallinity, instability to oxidation, and tissue penetration are the main drawbacks that require to be addressed in the design of advanced micro- and nano-materials. In this last section, we will report the most diffused approaches for fabricating formulations and active biomaterials for the treatment of skin disorders. The main strategies used for this purpose are schematized in Figure 4.

### 4.1. Micro and Nano-Particles

Many hydroxycinnamic acid derivatives have low aqueous solubility, poor stability, and slow transport across biological membranes, which result in low bioavailability. One way to improve their solubility and dissolution rates is to encapsulate them in drug delivery systems such as micro- and nanoparticles, liposomes, vesicles, cocrystals, and micelles.

For example, CinAc-loaded transfersomes were designed as transdermal delivery vectors [199]. These elastic liposomes are able to withstand high deformation, thanks to their phospholipidic structure, modified with a so-called edge activator, a single-chain surfactant whose role is to destabilize the lipid bilayer, thereby increasing its flexibility and circumventing the transportation across the stratum corneum. Aitipamula et al.[200] produced cocrystals of FA with urea, nicotinamide, and isonicotinamide, and dispersed them in oleogel formulations. The cocrystal systems demonstrated enhanced solubility in water, stability, and penetration into skin membranes with respect to pure FA. Suzuki et al. [201] developed poly lactic co-glycolic acid (PLGA) nanoparticles encapsulating CGA and reported that skin permeability and prolonged effects of the encapsulated ingredient were enhanced. Moreover, CGA has been previously identified as a compound that stimulates the production of type XVII collagen and its effects have been confirmed by in vitro experiments with epidermal keratinocytes. However, CGA solution could not promote the production of type XVII collagen due to its degradation in the medium, while in contrast, by applying CGA-loaded PLGA nanoparticles, the mRNA of type XVII collagen and the production of collagen were significantly increased. Recently, Ammar et al. [202] showed the encapsulation of *Posidonia oceanica* seagrass extracts in gelatin nanoparticles, which were fabricated to protect their phenolic acids content (including 4-hydroxybenzoic acid, ferulic acid, *p*-coumaric acid, and caffeic acid), control their release, and enhance their therapeutic activity. Chhabra et al. [203] fabricated and successfully evaluated, both in vitro and in vivo, RA-loaded chitosan (CH) encapsulated graphene nanoparticles (RA-CH-G-NPOs), with increased antimicrobial property and wound healing capacity. Indeed, wound contraction, cell adhesion, epithelial migration, and high hydroxyproline content were improved compared to plain RA and controls by histopathological evaluations. Another suitable strategy for treating diabetic chronic wounds was proposed by Alberti et al. [204]. They showed the potential, both in vitro and in vivo, of polyphenolic-rich propolis nanoparticles prepared by using an emulsification method with poloxamer and soy lecithin. Analyzing the propolis extract, the chromatogram highlighted a high content of phenolic acids derived from cinnamic acids (e.g., syringic acid, *p*-coumaric acid). The authors mentioned many advantages for this delivery system, such as an increment in stability, and suggested that propolis nanoparticles could accelerate the proliferative phase, collagen deposition, and angiogenesis in fibroblasts. Sguizzato and co-workers [205] loaded CA inside solid lipid nanoparticles using lipid fusion, hot homogenization, and ultrasonication, producing a nanoparticulate gel by adding poloxamer 407 and hyaluronic acid to the nanoparticle dispersion. The final product exhibited an eightfold slower CA diffusion compared to the aqueous dispersion when investigated by Franz cells. The CA anti-oxidant effect has also been evaluated ex vivo on human skin explants exposed to cigarette smoke, suggesting a protective role exerted by the nanoparticles, particularly on oxidative stress. Carbone et al. developed nanostructured lipid carriers (NLC), by using the phase inversion temperature method, for the combined delivery of FA and *Lavandula* essential oils, whose beneficial effects in wound healing processes have been widely reported [206]. The results suggested a potential combined effect of the co-delivered anti-oxidant FA and *Lavandula* essential oils in promoting fibroblast proliferation and migration. Interestingly, the free FA at the same tested concentration was not able to promote wound closure, while drug encapsulation in NLC allowed a controlled and slower FA release. Recently, a new class of liposomes named trans-ethosomes was used by Rodriguez-Luna et al. [207] to develop RA-loaded formulations. The authors showed the anti-inflammatory activity of this novel delivery system on IMQ-induced psoriasis-like skin inflammation in mice. In vitro release profiles demonstrated sustained behavior due to the entrapment of RA into the vesicles. In addition, treatment with RA formulations resulted in a significant reduction in the punch oedema volume as well as in the TNF-α and IL-6 levels. Similar results were obtained by Singh et al. [208], who formulated RA-loaded phyto-vesicles using soy lecithin and cholesterol in order to decrease the low solubility and instability of RA (Figure 4b). Optimized phyto-vesicles showed significant modulation of TNF-α, IL-1β, and IL-6 when compared to the control group in a TPA-induced ear inflammation model in Swiss albino mice. Moreover, biological assays showed that the equivalent dose of optimized phyto-vesicles possessed better anti-inflammatory activity compared to pure RA. This result may be attributed to the increased solubility of the active ingredient in the lipid phase, which provides easy penetration of the drug into the tissues.

### 4.2. Fibers

Electrospinning is the most common technique used for the fabrication of polymeric fibers with a range of diameters varying from few nanometers to some micrometers. For this reason, this technology is the optimal one for the treatment of skin wounds. Indeed, it allows the production of a three-dimensional scaffold able to mimic the skin ECM, promoting the attachment and the proliferation of the cells involved in the wound healing process (e.g., fibroblasts and keratinocytes) [209,210]. The electrospun process consists of an articulate setup composed of a high voltage power supply, a syringe with a capillary needle, a syringe pump, and a conductive collector [211]. A polymeric solution is loaded inside the syringe and pumped out from the tip of the needle to form a spherical drop by superficial tension. Meanwhile, an electric field is generated between the two electrodes of different charges, placed on the needle tip and on the collector. This potential difference promotes the formation of an excess of charges on the surface of the droplet, changing its form to a conical one (the so-called Taylor cone). At this point, if the voltage force exceeds the superficial tension, the polymeric solution can be ejected on the collector [212].

Often, the addition of bioactive molecules in the polymeric solution is needed for the fabrication of drug delivery scaffolds able to tune the release of the pharmaceutical agents in time to facilitate and speed up the healing process in the wound area [209]. Many studies in the literature are focused on the electrospinning of natural and synthetic polymer solutions loaded with hydroxycinnamic acids and derivatives, highlighting their pharmaceutical activity in treating skin injuries. For example, Kossyvaki, Suarato et al. [213] recently designed a composite electrospun matrix based on wool-recovered keratin and polyvinylpyrrolidone (PVP) loaded with different amounts of cinnamon essential oil for the treatment of skin burns caused by UVB exposure (Figure 4c). The biocomposite presented anti-oxidant properties (Figure 4f), cell biocompatibility towards human primary fibroblasts, and antimicrobial activities against *Staphylococcus aureus*, *Escherichia coli* and *Pseudomonas aeruginosa*. More interestingly, upon contact with the UVB-irritated skin, the keratin–cinnamon essential oil patch downregulated the expression of the inflammatory cytokines IL-1β and IL-6 of 7 and 5 folds, respectively, and mitigated the redness of the skin. Caffeic acid has been widely incorporated in a plethora of fibrous biomaterials. Ignatova et al. [214] successfully fabricated poly(3-hydroxybutirrate)/CA fibrous mats, which exerted antimicrobial and immune-stimulating activities toward spleen lymphocytes and peritoneal macrophages, resulting in a suitable material for the treatment of inflammatory skin diseases. In another work, the same research group incorporated the CA derivate CAPE in electrospun microfibers composed of poly(3-hydroxybutirrate) and PVP, demonstrating a good anti-oxidant activity and their ability to inhibit the adhesion of pathogens [149]. Similarly, CA and cyclodextrin were encapsulated in poly (vinyl alcohol) nanofibers, increasing the resulting antimicrobial activity [215]. Poly(ε-caprolactone) fibrous mats loaded with CA were able to stimulate the proliferation and spreading of normal human dermal fibroblast-neonatal (NHDF-neo) cells and showed high antimicrobial activity [216]. Chuysinuan et al. chemically modified the surface of poly(L-lactic acid) (PLA) fibrous mats with CA, obtaining a non-cytotoxic 3D scaffold presenting a good ability to support the human dermal fibroblast adult (HDFa) cells growth and a high anti-oxidant activity [217].

Poly(2-hydroxyethyl methacrylate) (pHEMA) nanofibers incorporating syringic acid were successfully produced by the electrospinning technique [97]. It was demonstrated that SA retained its anti-oxidant activity after incorporation into the pHEMA nanofibers and that the encapsulation can delay to a great extent its degradation induced by environmental factors. Adomavičiūtė et al. studies aimed to formulate electrospun fast-dissolving wound dressing mats containing propolis ethanolic extract (PEE, rich in coumaric, ferulic, caffeic, and vanillic acids) and silver nanoparticles. Produced electrospun nano/microfiber mats made with PVP or PLA were evaluated by studying their structure, dissolution rate, the release of propolis phenolic compounds and silver nanoparticles, and antimicrobial behavior. The PEE-matrices maintained the viability of HaCaT cells, while the antimicrobial activity tests confirmed the ability of these biocomposites to inhibit the growth of selected microorganisms [218,219]. Vatankhan et al. [220] developed a cellulose acetate nanofibrous mat as a carrier for the delivery of RA in order to treat injuries associated with inflammation. They showed that RA-loaded cellulose acetate nanofibers were able to exhibit the same bioactivities of free RA, including anti-inflammatory, anti-oxidant properties, and biocompatibility towards epithelial cells.

Sandova-Herrera et al. [221] used CGA encapsulated in PVA and PVA blended with poly-γ-glutamic acid (γ-PGA) electrospun nanofiber mats for the sustained release of an anti-diabetic compound with potential medical application in the treatment of the diabetic foot. CGA was successfully incorporated into PVA or PVA/γ-PGA nanofiber mats and released to a buffer phosphate media in a sustained manner for more than 200 h.

The high voltage and the strong shear stress involved in the electrospinning process might impair the bioactivity of the encapsulated drug. To circumvent this possible issue, coaxial electrospinning represents a valid alternative. This technique consists of the extrusion of two different polymeric solutions through a concentric needle to produce core-shell layered fibers [222]. Poornima et al. [223] used this setup to design a controlled release nanofiber scaffold using chitosan, a biocompatible polymer, in the core and poly-caprolactone, a synthetic polymer with good biocompatibility and biodegradability characteristics, as the shell. The hydrophilic core allowed the incorporation of FA and its controlled release in the wound bed.

### 4.3. Hydrogels and Scaffolds

Hydrogels are soft, 3D macromolecular networks that can take up a high amount of liquid, thus undergoing a rapid swelling in a water-like environment. Depending on the polymeric chains in the 3D arrangement, hydrogels can sustain intense deformability levels while keeping their overall shape. Thanks to this peculiar behavior and its similarity of the property of some body tissues, hydrogels have found several applications in the biomedical field as drug delivery platforms or substrates for cell seeding and growth [224]. Biologically active principles can be physically entrapped within the hydrogels, protected and stabilized during the storage in the dry state, and then released by diffusion or matrix erosion when in contact with water or moisture-containing milieu (e.g., the wound exudate). Recently, functionalization moieties have been included to obtain stimuli-responsive system, able to finely tune their structures and properties (degradability, mechanical resistance, hydrophilicity) upon a specific external trigger (e.g., pH, temperature, light, electric field), thus greatly extending the potentialities of this biomaterial platform [225].

Presenting a more defined architecture and 3D spatial organization, scaffolds serve the purpose of mimicking the extracellular matrix when a new tissue needs to be formed at a lesion site. Cells can be seeded onto the provisional matrix and let grow until the complete restoration of the tissue continuity is achieved. Scaffolds can be constituted of either synthetic polymers or macromolecules of natural origin such as protein and polysaccharides, and, depending on their composition and fabrication method (e.g., self-assembly, 3D printing, lyophilization, fiber spinning) can present different mechanical properties and degradability [226]. Usually, bioactive compounds are loaded within the scaffolds to favor cell adhesion and boost the tissue regeneration properties. The naturally-derived molecules presented in this review have been either loaded into hydrogel-like constructs or encapsulated within a polymeric scaffold matrix to improve their shelf-life and enhance their therapeutic ability.

Das et al. [227] prepared hydrogel formulations containing FA-loaded nanocapsules to increase its stabilization. In this study, they demonstrated how encapsulating FA in a low pH hydrogel was able to preserve the active agent’s stability and increase its ability to permeate into the different layers of the skin, even deep ones, without interfering with the skin microbiome. Also, Sivakumar and colleagues [228] developed a biomaterial composite presenting a synergy action between FA and the keratin/chitosan/fibroin scaffold. Ferulic acid was encapsulated into microspheres that were embedded within keratin scaffolds, thus further modulating the active molecule release profile. The authors demonstrated a combination of different properties of the various components which exerted an antimicrobial action and promoted wound healing.

CA was conjugated with a gelatin injectable hydrogel to improve the overall biomaterial rheology, mechanical stability, antimicrobial and anti-oxidant activities [229] (Figure 4d). Also, fibroblast cells were able to adhere and proliferate inside the hydrogel 3D structure (Figure 4g). Similarly, Rao et al. [230] conjugated CA with gelatin to fabricate an injectable hydrogel that supports the survival and proliferation of human gingival mesenchymal stem (HGMS) cells. CA was also successfully loaded inside chitosan and collagen hydrogel, improving their anti-oxidant proprieties significantly [205].

Budhiraja et al. [231] investigated the anti-acne ability of RA by encapsulating it into a niosomal gel and by testing it in vivo on Swiss albino mice. Both plain drug and niosomal formulations inhibited inflammation processes, but only the niosomal gel reduced the bacterial multiplication rate 4 days after application due to its prolonged release. As a promising wound healing scaffold, Azadmanesh et al. [232] fabricated a highly porous three-dimensional-printed scaffold using PLA. The core of the structure was composed of hyaluronic acid, copper carbon dots, RA, and chitosan hydrogel. This nanocomposite was applied on wound areas in mice and proved to be a promising scaffold that highly accelerates the process of skin regeneration.

PVP was also employed to entrap both PCA and FA in micro-fibers produced via the electrospinning method [41]. Later, the polymer was thermally annealed, leading to innovative fibrous hydrogels. During the procedure, the PCA and FA resulted not affected and still intensely active both in in vivo mice model, reducing statistically the inflammatory mediators MMP-9 and GPX-1, and in an ex vivo human skin model that simulates a chronic wound condition, reducing after 3 days both IL-6 and IL-8 levels. Of note, the FA resulted more active than PCA. The biomaterials were also able to promote and not affect the wound healing process of the human skin.

Finally, cross-linked, hyperbranched polyesters made of glycerol, aconitic acid, and CinAc were synthesized as scaffold substrates for stem cells differentiation and collagen production [233].

Therefore, combining the bioactivity of the above-mentioned natural extracts with the 3D structure of a macromolecular matrix constitutes a promising strategy to design effective targeted therapies for acute, chronic, and burn lesions healing.

### 4.4. Films and Creams

Thin-film patches have been widely used in the last years as suitable biomaterials for skin disorders. Commonly produced by solvent casting and spin-, rod- or roll coating methods, they can ensure a full cover of the area and an in situ delivery of active compounds [234,235]. Both mono- and multi-layers films have been designed based on synthetic or natural polymers [236,237,238,239]. These formulations can absorb exudate and physically protect the missing tissue from microorganisms and dehydration in case of skin loss, acting as a shield. They can also be fully transparent, allowing the check of the underlying area and/or occlusive agents enhancing the skin hydration, a helpful parameter in dermatitis and psoriasis [240,241,242].

Despite being widely present in the literature, only a few works have been found using this type of film formulation for the herein discussed phenolic acids.

PCA has been included in a PVP matrix as a potential transparent film wound dressing [243] (Figure 4e). PCA could change the peculiar characteristics of PVP, high water solubility, and poor mechanical properties, acting as a plasticizer and interacting with PVP through H-bonds, and allowing a controlled PCA release. At the same time, the PVP could interrupt the inter-intramolecular interactions of PCA avoiding crystallization phenomena during the film production and increasing the final amount of the loaded drug. Moreover, when dispersed in the PVP, the degradation temperature of PCA shifted to higher temperatures, enhancing the thermal stability of the phenolic acid. Finally, the ameliorated films resulted in having anti-bacterialefficacy against *E. coli* (Figure 4h) and *S. aureus* and statistically reducing the MMP-9, a key target in diabetic chronic wounds [244], in in vivo mice models, suggesting a suitable activity of the produced biomaterials. Furthermore, PCL-PCA copolymers at the different ratios between the two compounds were synthetized by Contardi and Alfaro et al. [245]. After the synthesis, the copolymers were produced and characterized in the form of films. The final biomaterials resulted in having stronger anti-oxidant activity, suitable barrier, and mechanical properties with respect to the pristine PCL and PCA. The copolymerized PCA thermally degraded at 445 °C, suggesting superior thermal stability in the copolymer with respect to pure PCA. Finally, the copolymers resulted in hemocompatible, biocompatible, and anti-bacterialcharacteristics, meaning that these new copolymers can be employed to design sutures or biomaterials for skin regeneration.

On the other hand, more studies describe creams and ointments loaded with hydroxycinnamic acids and derivatives. Being dispersed in a more hydrophobic medium, such formulations can help overcome the solubility problems typical of the phenolic compounds and enhance their penetration into the skin. Žilius et al. [246] studied the penetration of vanillic, coumaric, caffeic, and ferulic acids into epidermis and dermis from designed semisolid vehicles, such as ointment, cream, and hydrogel. Results of the study demonstrated that the ability of the phenolic acids to penetrate the skin epidermis and dermis was affected by the delivery systems. In particular, VA presented relatively high penetration through the epidermis into the dermis, where this compound was found to be more concentrated irrespectively from the formulation used. PCA and FA diffused into the skin using the cream and the ointment but not when loaded into the hydrogel; both compounds were uniformly distributed between epidermis and dermis. Lastly, CA slowly penetrated the epidermis only when dispersed in a cream formulation, and it was not detected in the dermis.

Furthermore, Seo et al. [247] evaluated a PCA-based cream on a volunteer patient as therapy for the erythema and pigmentation after UV exposure, taking advantage of the anti-oxidant properties of PCA. Kuba et al. [248] investigated the effect of a 10% RA topical cream based on white beeswax on a rat experimental wound model. The authors reported an enhancement in the wound repair rate in comparison with the results obtained in the control group and in animals treated with a dexpanthenol 5% cream.

Using oil-in-water microemulsions consisting of NaCl solution, isopropyl myristate, Tween 80, and ethanol, Kitagawa et al. [249] assessed the topical delivery of CGA. In particular, intradermal delivery of CGA to guinea pigs during exposure to UVB reduced photo-oxidation damage of the skin (e.g., erythema formation). Conventional topical formulations of CGA might not be able to produce the desired protective effect after long exposure to sunlight since CGA has a lower degradation half-life and a higher rate of distribution and elimination [250]. To address this problem, Bhattacharyya et al. prepared a novel topical sustain-release CGA formulation based on phospholipids. The complex was prepared and evaluated against oxidative stress produced in the rat skin due to UVA exposure. Compared to the conventional formulation, the phospholipid formulation exerted improved protection after 4 h of topical application [251]. To achieve efficient skin delivery of polyphenols, Yutani et al. [252] prepared a novel oil-in-water type microemulsion (MESL) using sucrose laurate as a surfactant and ethanol, isopropyl myristate, and water as other components. The authors examined its usefulness by conducting in vitro studies on CGA skin delivery using Yucatan micropig skin. CGA delivery efficiencies into the skin induced by MESL at 40 h after application were approximately 6-fold higher in the epidermis and 3.5-fold higher in the dermis. Chen et al. evaluated the beneficial effect of topical chlorogenic acid treatment on excision wounds in Wistar rats. A 1% *w*/*w* chlorogenic acid or silver sulfadiazine ointment was applied topically once a day. On the 15th post-surgery day, the wound contraction demonstrated that the CGA ointment had a potent wound healing ability. The treated rats showed increased rates of epithelialization, higher TNF-α levels during the inflammatory phase of wound healing, upregulated transforming growth factor-β1, and collagen IV synthesis. Their results also indicated that CGA possesses potent anti-oxidant activity by increasing superoxide dismutase, catalase, and glutathione levels while decreasing lipid peroxidation [253].

## 5. Conclusions and Future Perspectives

In the last years, hydroxycinnamic acids and derivatives have been investigated and exploited due to their remarkable biological properties, making them an attractive platform for various cutting-edge applications, from food packaging to electrochemistry and biomedicine.

Skin disorders are characterized by different issues to be addressed, such as permanent inflammation, harmful free radicals, and microorganisms’ infections. These natural molecules have proven to be potential alternative drugs, ensuring effective actions against several pharmacological targets for the most diffused skin disorders. Currently, most of the published researches have been focused on exploiting these phenolic acids for wounds and UV-induced skin injuries. On the other hand, psoriasis and dermatitis are still unexplored fields for the major part of these compounds. Indeed, only a few studies described the effect of FA and RA for these pathological conditions. Further studies are required to investigate the capability of these compounds to act as supportive therapy in combination with the standard treatments under pharmacological and toxicological aspects.

Currently, a plethora of different methodologies have been explored to entrap these phenolic acids, mostly based on micro- and nano-formulations such as nanoparticles, nanofibers, hydrogels, thin films, and creams. These delivery systems revealed the possibility of overcoming the hydroxycinnamic acids and derivatives drawbacks, usually connected with poor solubility in water, stability, and limited skin penetration. However, the most emerging delivery systems such as microneedles, 3D-printed, and mycelium-based scaffolds have not been evaluated yet to encapsulate and release these compounds, suggesting future exciting perspectives in this direction. For instance, microneedles systems could allow an easier delivery and a deeper penetration of the phenolic acids into the dermis, overcoming the epidermal barrier and potentially ameliorating the efficacy in conditions where the skin thickness is altered such as psoriasis and dermatitis[254]. Furthermore, the scalable and rapid production of 3D-printed biomaterials[255] loaded with these phenolic compounds could be helpful in developing scaffolds for the case of large-area skin loss, ensuring a quick and suitable application as first-aid products. Finally, mycelium-based materials are blooming as a new generation of all-natural biomaterials. Several strains of fungal mycelia have been proven to release phenolic compounds[256], and recently, they have been evaluated as potential scaffolds for the proliferation of human fibroblast and keratinocyets cell lines [257,258]. These outcomes make them a future promising tool for the production of delivery systems of phenolic acids.

In a scenario where the demand for alternative drugs and sustainable biomaterials is increasing daily, as a result of the large number of people affected by a variety of skin disorders, this class of molecules constitutes an up-and-coming platform for future skin treatment and wound management.

## Abbreviations

AA	Arachidonic acid
AD	Atopic dermatitis
AP-1	Activator Protein-1
CA	Caffeic Acid
CAPE	Caffeic Acid Phenethyl Ester
CH	Chitosan
CinAc	Cinnamic Acid
CGA	Chlorogenic Acid
COX-2	cyclooxygenase-2
DMSO	Dimethyl Sulfoxide
DNCB	2,4-Dinitrochlorobenzene
DNFB	1-Fluoro-2,4-dinitrobenzene
DPPH	2,2-Diphenyl-1-picrylhydrazyl
ECM	Extracellular Matrix
EGFR	Epidermal Growth Factor Receptor
FA	Ferulic Acid
GPX	Glutathione Peroxidase
GRAS	generally recognize As Safety
GSH	glutathione
HaCaT	Human Dermal Keratinocyte
HDFa	Human Dermal Fibroblast adult
ICAM-1	Intracellular Adhesion Molecule-1
IFN-γ	Interferon-gamma
IgE	Immunoglobulin E
IL-10	Interleukin-10
IL-12	Interleukin-12
IL-13	Interleukin-13
IL-17	Interleukin-17
IL-17A	Interleukin-17 A
IL-17RA	Interleukin-17 receptor A
IL-1β	Interleukin-1β
IL-2	Interleukin-2
IL-23	Interleukin-23
IL-31	Interleukin-31
IL-4	Interleukin-4
IL-5	Interleukin-5
IL-6	Interleukin-6
IL-8	Interleukin-8
IMQ	Imiquimod
IRAK1	Interleukin-1 Receptor Associated Kinase 1
IRAK4	Interleukin-1 Receptor Associated Kinase 4
JAK	Janus Kinase
LPS	Lipopolysaccharide
MAPK	Mitogen-activated protein kinase
MDCK	Madin-Darby Canine Kidney
MESL	oil-in-water-type Microemulsion
MMP-1	Matrix Metalloproteinase-1
MMP-3	Matrix Metalloproteinase-3
MMP-9	Matrix Metalloproteinase-9
MyD88	Myeloid Differentiation Factor 88
NADH	Nicotinamide adenine dinucleotide hydride
NFE2	Nuclear Factor Erythroid 2
NF-κB	Nuclear Factor kappa-light-chain-enhancer of activated B cells
NHDF-neo	normal human dermal fibroblast-neonatal
NLC	Nanostructured Lipid Carrier
NLRP3	Nucleotide-binding oligomerization domain
NO	Nitric Oxide
NRF2	nuclear factor erythroid 2–related factor 2
PCA	*p*-coumaric acid
PCL	Poly *ε*-caprolactone
PGE2	Prostaglandin E2
pHEMA	poly(2-hydroxyethyl methacrylate)
p-IκB-α	nuclear factor of kappa light polypeptide gene enhancer in B-cells inhibitor alpha
PIK3/AKT	Phosphoinositide 3-kinases; alfa serine/threonine-protein kinases
PLA	Poly Lactid Acid
PLGA	Poly Lactic Co-Glycolic Acid
PPARγ	Peroxisome proliferator-activated receptor γ
PTP- κ	Protein tyrosine phosphatases-kappa
PVP	Polyvinylpyrrolidone
RA	rosmarinic acid
ROS	reactive oxygen species
SA	syringic acid
SOD	Superoxide dismutase
STAT	signal transducers and activators of transcription
Th2	T helper type 2 cells
Th17	T helper type 17 cells
TLR-3	Tool-Like Receptor-3
TLR-4	Tool-Like Receptor-4
TNFα	Tumor Necrosis Factor α
TPA	12-O-tetradecanoylphorbol-13-acetate
VA	vanillic acid
γ-PGA	Poly-γ-Glutamic Acid
